# MRI directed multidisciplinary team preoperative treatment strategy: the way to eliminate positive circumferential margins?

**DOI:** 10.1038/sj.bjc.6602947

**Published:** 2006-02-07

**Authors:** S Burton, G Brown, I R Daniels, A R Norman, B Mason, D Cunningham

**Affiliations:** 1 Royal Marsden Hospital, Downs Road, Sutton, Surrey SM2 5PT, UK

**Keywords:** magnetic resonance imaging, rectal cancer, chemoradiotherapy, multidisciplinary team, circumferential resection margin

## Abstract

Histopathological audit of positive circumferential resection margins (CRMs) can be used as a surrogate measure of the success of rectal cancer treatment. We audited CRM involvement in rectal cancer patients and the impact of the multidisciplinary team (MDT) on implementing a magnetic resonance imaging (MRI)-based preoperative treatment strategy. Data were collected on all newly diagnosed rectal cancer patients treated in our network between January 1999 and December 2002. Data were analysed for MRI prediction and histopathological assessment of CRM together with the MDT meeting treatment decisions. The CRM+ve rate of those discussed at MDT *vs* those not discussed were compared. We re-audited the CRM+ve rates 1 year after introducing a policy of mandatory preoperative MRI-based MDT discussion. Of the 298 patients diagnosed with rectal cancer, 39 (13%) were deemed palliative, 178 underwent surgery alone and 81 underwent neoadjuvant therapy. Of these, 62 out of 178 patients underwent surgery alone without MRI-based MDT discussion resulting in positive CRM in 16 cases (26%) as compared to 1 out of 116 (1%) in those patients with MDT discussion of MRI. Overall CRM+ve rate in all nonpalliative patients with or without MDT discussion was 12.5% (32 out of 256), significantly lower than the <20% rate (*P*<0.001) quoted in national guidelines. Re-audit in 98 consecutive patients following a change of policy produced a lower CRM+ve rate of 3% (1 out of 37) for all surgery alone patients and an overall CRM+ve rate of 7% (5 out of 70). In conclusion, MDT discussion of MRI and implementation of a preoperative treatment strategy results in significantly reduced positive CRM in rectal cancer patients.

The Colorectal Cancer Multidisciplinary Team (MDT) is composed of specialist surgeons, clinical and medical oncologists, radiologists, histopathologists and specialist nurses. The MDT should implement an agreed treatment strategy in rectal cancer patients based on nationally accepted guidelines with the aim of standardising and improving outcomes (2001; 2004). Currently, there is no published data to support the central role of the MDT discussion in effecting improved outcomes.

The main outcome measures of rectal cancer treatment have traditionally been the local recurrence rates, the development of distant metastases and overall survival. More recently, the circumferential resection margin (CRM) has been identified as an indicator of the quality of surgery within a unit, and there is now good evidence to relate the CRM status to improved outcomes (Birbeck *et al*, 2002; Nagtegaal *et al*, 2002). A positive CRM can be defined as the histological identification of tumour within 1 mm surgical resection margin. The presence of a positive CRM has been shown to correlate with an increasing incidence of local recurrence, systemic failure and poor survival (Hall *et al*, 1998; Delaney *et al*, 2002; Nagtegaal *et al*, 2002). However, some published data have defined a positive CRM as the presence of tumour within 2 mm of the surgical resection margin as a more accurate predictor of poor survival (Beets-Tan *et al*, 2001; Nagtegaal *et al*, 2002; Marijnen *et al*, 2003).

In addition to CRM status, increasing depth of spread has also been identified as a poor prognostic factor based on an analysis of 853 patients in the Erlangen Registry of Colorectal Carcinomas. In this study, the prognostic value of the depth of invasion beyond the bowel wall reported that extramural spread ⩾5 mm resulted in a markedly reduced 5-year survival of 54% because of systemic failure as compared to 85% in those with extramural spread <5 mm (Merkel *et al*, 2001). Other important poor prognostic features include N2 nodal disease (four or more involved nodes), T4 disease and extramural venous invasion (Shepherd *et al*, 1995).

High spatial resolution magnetic resonance imaging (MRI) has achieved good accuracy in the preoperative prediction of positive CRM, depth of extramural spread and other poor prognostic features suggestive of locally advanced disease (Brown *et al*, 2003). Such imaging can identify patients at high risk of local and systemic failure in whom a preoperative treatment strategy could be beneficial.

There is good evidence from the Dutch TME Trial that preoperative short-course radiotherapy does not compensate for positive resection margins (Marijnen *et al*, 2001; Marijnen *et al*, 2003). Therefore, it has been our policy to proceed with surgery alone if MRI shows favourable prognostic features and to employ neoadjuvant chemoradiotherapy as a means of inducing regression in locally advanced rectal cancers (Janjan *et al*, 1999; Chau *et al*, 2003). The effectiveness of this approach can be judged by auditing the histological CRM−ve rates. Such rates can reflect the success of the MDT process in addition to the effectiveness of the implementation of established guidelines.

The aim of this study was to audit the CRM+ve rates in rectal cancer patients treated within our network after MDT discussion of MRI and implementation of an appropriate preoperative treatment strategy.

## PATIENTS AND METHODS

A database was compiled of all consecutive biopsy-proven primary rectal cancer patients treated within our network, between January 1999 and December 2002. The network consists of four hospitals and six specialist colorectal surgeons. The data were collected from the preoperative MDT meeting minutes, histopathology records and MRI reports. For each case, it was noted whether an MRI had been performed and whether the case had been discussed preoperatively at an MDT meeting. Our network staging policy required a pelvic MRI and abdominal computerised tomography (CT) with either CXR or CT thorax to determine the presence of metastatic disease. Patients were deemed palliative if they had metastatic disease unsuitable for resection or significant comorbidity that precluded any therapeutic intervention.

**Figure 1:**
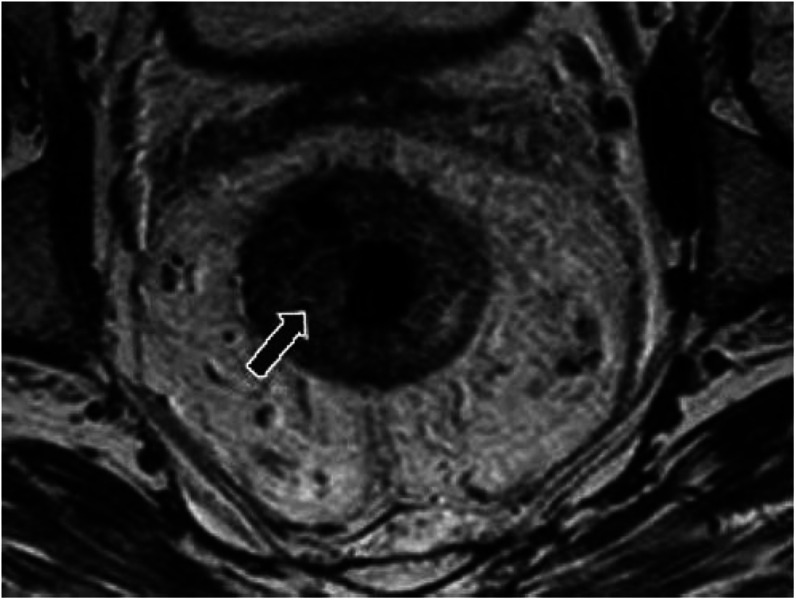
Example of a good prognosis tumour (Group 1). Axial T2-weighted high-resolution image showing an annular tumour with no evidence of extramural spread, no suspicious lymph nodes and clear potential resection margins.

Magnetic resonance imaging was used to categorise nonpalliative patients into three groups according to prognostic features:

*Group 1*. The primary tumour showed good prognostic features with potentially negative CRM. Such features included T1 and T2 tumours (except those low tumours described in Group 3) and T3 tumours with a depth of extramural spread less than 5 mm together with less than four nodes involved with tumour and no evidence of extramural vascular invasion (Figure 1).

**Figure 2:**
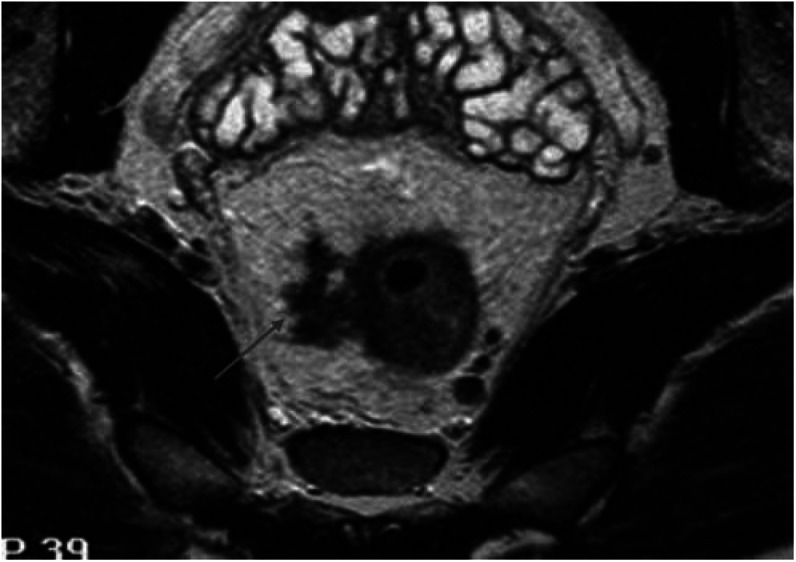
Example of a bad prognosis tumour showing EMV but CRM−ve (Group 2). Axial T2-weighted image showing tumour extending into an extramural vein; the distance of tumour to the potential circumferential margins, however, is >1 mm, so the margins are considered safe.

*Group 2*. The primary tumour showed any poor prognostic features with potentially negative CRM. Such poor prognostic features included peritoneal involvement (T4 disease), T3 tumours with extramural depth of spread ⩾5 mm, presence of extramural vascular invasion and four or more involved lymph nodes (Figure 2).

**Figure 3:**
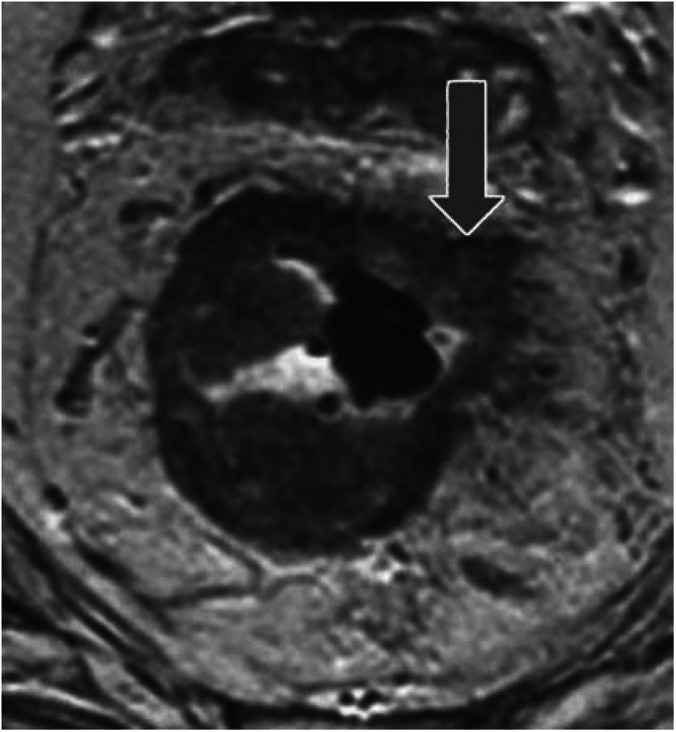
Example of a potential CRM+ve tumour (Group 3). Axial T2-weighted image depicting and annular infiltrating tumour. Tumour extend to the mesorectal fascia anteriorly (arrow), the potential circumferential resection margins are therefore considered involved.

*Group 3*. The primary tumour showed potentially positive CRM (including full thickness T2 tumours arising below the origin of the levator ani muscles), which is known to be associated with a higher rate of margin positivity and therefore a worse outcome (Table 1) (Figure 3). (During abdomino-perineal resection, there is a tendency for the plane of dissection to ‘waist’ below the level of the levators and a wide cylindrical dissection may be difficult to achieve. This has translated into unacceptable levels of local recurrence and involved margins. Hence, very low T2 tumours have been incorporated into the worst prognosis treatment group.)

Margin positive disease was defined on MRI as tumour extending to within 1 mm or beyond the mesorectal fascia which forms the surgical resection margin in total mesorectal excision (TME) surgery.

Our local treatment stratification was based on evidence of:

- Five-year survival rates of 85% for rectal cancer patients with T3 tumours <5 mm depth of extramural spread *vs* 54% survival for cases with T3 ≥5 mm spread (Merkel *et al*, 2001).
- Peritoneal involvement is a known poor prognostic factor for rectal cancers (Shepherd *et al*, 1995).
- Increasing percentage of nodal disease reduces overall survival in colorectal cancer (Stocchi *et al*, 2001).
- EMV has been shown to be an independent poor prognostic factor in colorectal cancer (Conroy *et al*, 1994; Mulcahy *et al*, 1997).

### MRI technique

Only T2-weighted sequences were used. Magnetic resosnance imaging was performed on a 1.5 T scanner with a four-element pelvic phased array wrap-around surface coil in the primary hospital. All patients were imaged in the supine position. Neither intravenous antiperistaltic agents or contrast agents were administered. A coronal localising image was obtained to select axial and sagittal images with a T2-weighted FSE sequence (TR >3000 ms, TE=128 ms, ETL=16, 5 mm thickness, four signal averages, scan duration 3–5 min). The sagittal images were used to plan 3 mm oblique high spatial resolution axial images. The images were acquired in a plane orthogonal to the tumour and rectal wall using a T2-weighted FSE sequence (TR>3000, TE=128 ms, 256 × 256 matrix, ETL =16, FOV=16–18 cm). Depending on the length of tumour, scan duration was between 6 and 12 min.

### Chemoradiation regime

Patients received protracted venous infusion 5-fluorouracil (5-FU) (300 mg m^−2^ day^−1^ for 12 weeks) with mitomycin C (7 mg m^−2^, i.v. bolus every 6 weeks). Starting on week 13, 5-FU was reduced to 200 mg m^−2^ day^−1^ and concomitant pelvic radiotherapy 45 Gy in 25 fractions was commenced followed by 5.4–9 Gy boost to tumour bed. Surgery was planned 6 weeks after chemoradiation (Chau *et al*, 2003).

Any patient deemed eligible for neoadjuvant treatment on the basis of poor prognostic disease or involved margins but with contraindications to chemotherapy underwent long course radiotherapy (LRT) alone. As with the chemoradiotherapy group, surgical resection was planned 6 weeks following therapy. For the purposes of analysis, the LRT patients were included in the relevant neoadjuvant treatment groups on an intention to treat basis.

### Surgical technique

All surgical resections were performed with curative intent. The operations performed included Hartmann's procedure, high anterior resection, low anterior resection with TME and loop ileostomy and abdomino-perineal resection. The decision with regard to the most appropriate operation was based on site of tumour together with patient characteristics (including ano-rectal physiology if deemed necessary) and surgeon's preference.

### Histopathological technique

Histopathology examinations were performed according to the Royal College of Pathologists guidelines (Quirke and Williams, 1998). The CRM was defined as positive if tumour was within 1 mm of the surgical resection margin. In cases of complete histopathological response of tumour to neoadjuvant therapy, multiple representative axial slices were taken throughout the rectal specimen. T-stage, N-stage, CRM status and depth of extramural invasion were documented for the purposes of the study.

### Analysis

The CRM+ve rate of those discussed at MDT *vs* those not discussed were compared using the kappa test. The 95% confidence intervals (95% CI) were calculated using the binomial distribution. Circumferential resection margin rates were compared to national standards of <20% (2004) and published CRM+ve rates of 28% (Birbeck *et al*, 2002), using the binomial distribution.

### Closure of the audit loop

The results of the audit were presented to the multidisciplinary team and since then, it has been mandated that all rectal cancer patients and their MRIs be discussed by the MDT. A re-audit was conducted from October 2003 to July 2004, following the introduction of this new directive. The same treatment stratification groups were used as described above.

## RESULTS

**Figure 4:**
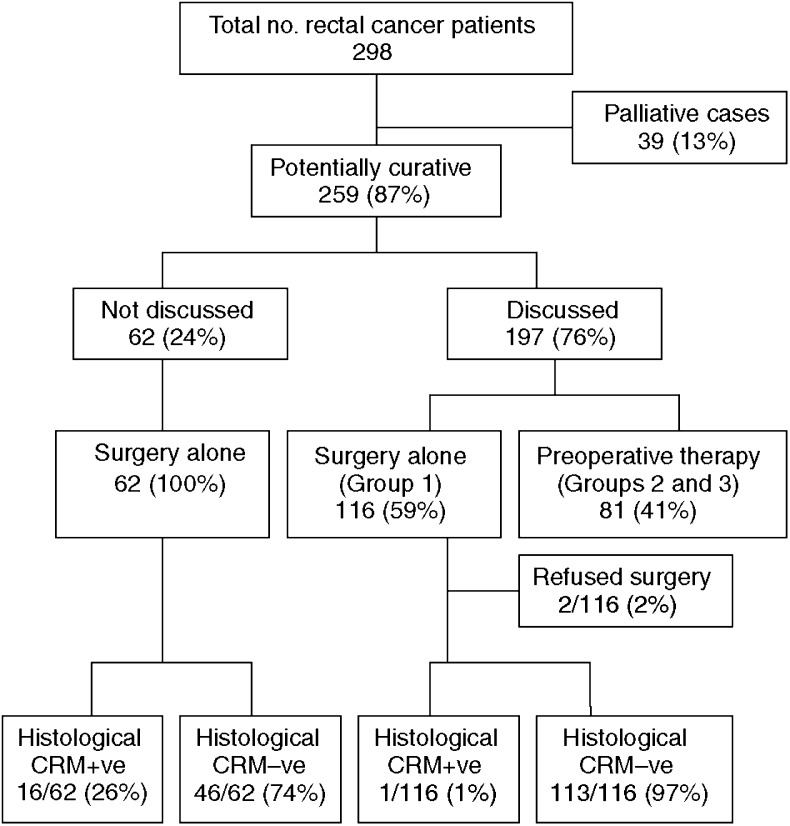
Actual treatment allocation.

Between January 1999 and December 2002, 298 patients diagnosed with biopsy-proven adenocarcinoma of the rectum were treated within the network. Median age was 67 years (range: 28–88 years) and 58% were male. Thirty-nine patients (13%) were classified as palliative either because of irresectable metastatic disease or significant comorbidity that precluded therapeutic intervention – not all these cases were discussed at MDT. Thus, 259 patients (87%) were identified as eligible for potentially curative therapy (Figure 4). One hundred and ninety-seven patients (76%) underwent preoperative discussion following an MRI and of these 81 (41%) were referred for preoperative neoadjuvant therapy on the basis of having poor prognostic features or a threatened or involved CRM (Groups 2 and 3). Of the 81 patients undergoing neoadjuvant treatment, six patients had contraindications to chemotherapy and therefore underwent LRT. All six of these patients proceeded to surgical resection.

The audit identified 62 out of 259 (24%) patients who proceeded to surgery alone without preoperative MDT discussion of MRI. In addition, 116 patients with good prognosis tumours were discussed preoperatively and deemed suitable for primary surgery. However, two of these patients refused surgery (Figure 4). Of the 62 patients undergoing surgery alone without an MDT discussion, the CRM+ve rate was 26% (95% CI=16.6–39.7%) (Figure 4). This contrasts with a 1% CRM+ve rate (1 out of 116) in those patients undergoing surgery alone after MDT discussion. Even the inclusion of the two patients who refused surgery as presumed CRM+ve data would still only increase the CRM+ve rate in this group to 3% (3 out of 116) (Figure 4).

**Figure 5:**
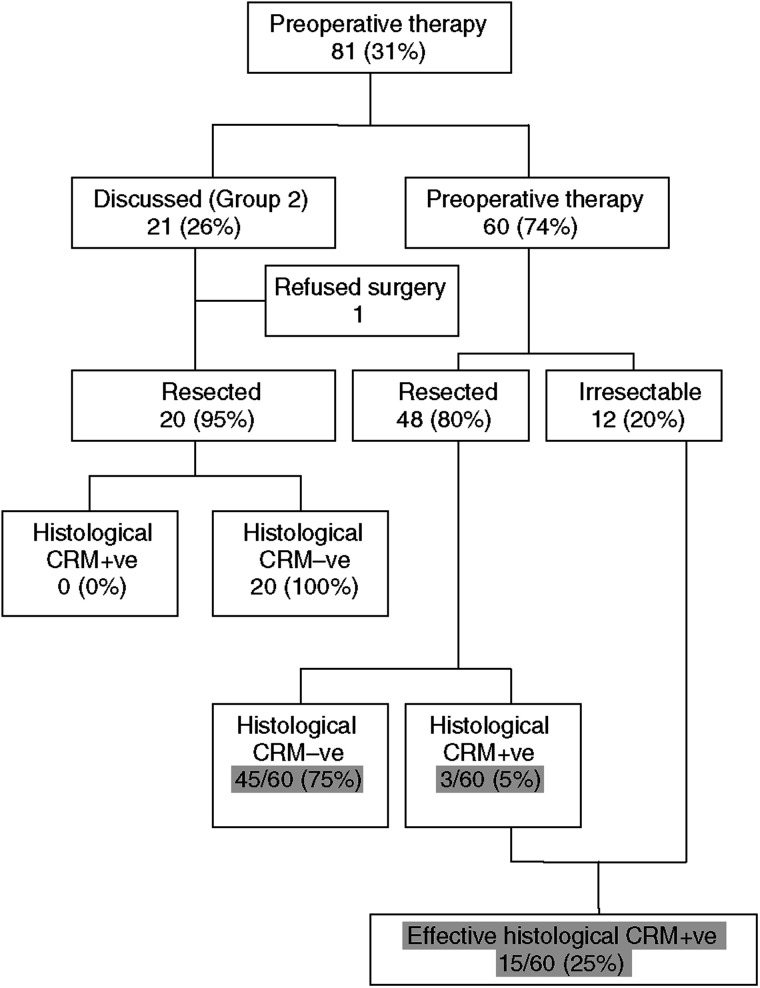
Histological CRM status in patients undergoing chemoradiotherapy.

Of the 81 patients undergoing neoadjuvant therapy, 21 were in Group 2 (Figure 5) including three patients who underwent LRT. One patient refused surgery after a complete response to preoperative neoadjuvant therapy as determined by MRI. There were no positive margins in Group 2 (Figure 5). Of the 60 patients in Group 3, the predicted margin positive group, only 12 patients (20 %) remained unresectable following completion of preoperative neoadjuvant therapy (two of these had received LRT only). Of the 48 patients (80%) who proceeded to resection, only three were CRM+ve following resection (Figure 5). Therefore, out of the 60 patients in group 3, 45 (75%) achieved a negative CRM.

MRI prediction of appropriate treatment group in the surgery alone patients was correct in 93% (106 out of 114). Of the incorrectly predicted patients, 6% (7 out of 114) had one or more poor prognostic feature identified histologically following resection. The remaining patient had a tumour perforation at the time of surgery, which could not have been predicted preoperatively.

Table 2 shows the MRI predicted prognostic groupings and the final actual prognostic groups as determined by histology. The predicted CRM+ve rate was 30% in patients undergoing MDT discussion of MRI. However, on the final histology following preoperative neoadjuvant therapy in Groups 2 and 3, there has been considerable downstaging with 152 (77%) of tumours demonstrating good prognostic features. This contrasts with Table 3, the histological staging of patients who did not undergo MDT discussion of MRI. Within this group, it can be seen that had these patients been discussed at MDT, it is likely that 32 (52%) would have been offered preoperative neoadjuvant therapy if the network guidelines had been followed, and a corresponding reduction in CRM positivity could have been anticipated. There was no significant difference between the predicted CRM positivity rate in the discussed group (60 out of 197, 30%) and the histological CRM positivity rate in the nondiscussed group (16 out of 62, 26%) (*P*=0.483) (Tables 2 and 3).

The CRM+ve rate for all patients undergoing surgery alone whether discussed or not was 9.5% (17 out of 180). Overall, there was a 2% CRM+ve rate (4 out of 182) in all resected patients discussed at MDT. If the 12 patients with irresectable disease were included together with the patients refusing surgery, the cumulative CRM+ve rate was 8% (16 out of 197). Therefore, the CRM+ve rate for all patients who underwent MDT discussion following an MRI (8%) is significantly lower than those patients not discussed (8 *vs* 26%) (*P*<0.001). These rates can be compared to national figures (2004) (8 *vs* <20%) (*P*<0.001). However, if we include those patients not discussed at MDT, the overall CRM+ve rate becomes 12.5% (32 out of 259), which is still well below recognised guidelines (2004).

**Figure 6:**
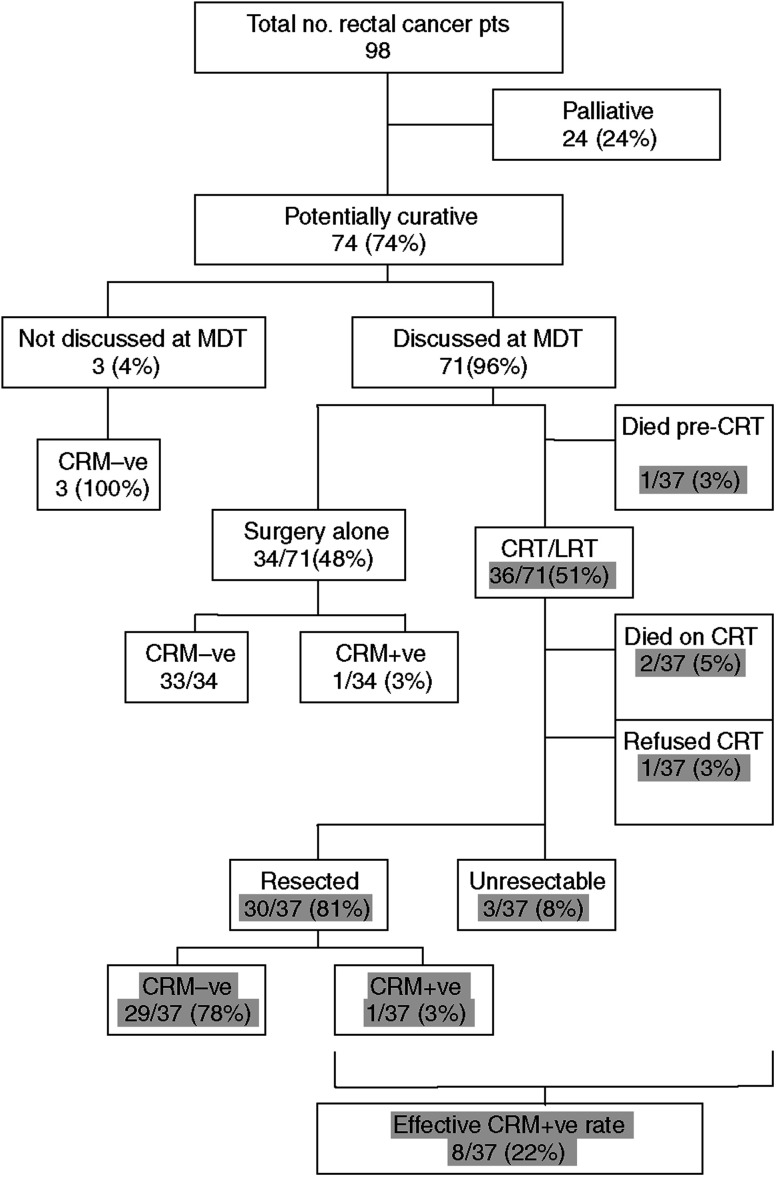
Flow chart of results of re-audit.

The re-audit (closure of the audit loop) identified 98 rectal cancer patients in a 10-month period from the same catchment area under the care of the original six surgeons. Twenty-four of ninety-eight patients (24%) were deemed palliative after MDT discussion on the basis of irresectable metastatic disease or profound co-morbidity (Figure 6). Of the 74 patients deemed potentially curative, only three patients (4%) did not undergo MRI or MDT discussion (Figure 6). All of these patients proved to be CRM−ve on histology. Seventy-one of seventy-four potentially curative patients did undergo MDT discussion of MRI. Thirty-four of these underwent surgery alone and of these, 33 were proven to be CRM−ve. Of the 37 patients selected for neoadjuvant therapy, one patient died of a pulmonary embolus following a defunctioning colostomy before commencement of preoperative neoadjuvant therapy. A further two patients died of cardiac problems during the initial chemotherapy and one patient refused treatment. Of the remaining patients, 30 underwent resection after preoperative neoadjuvant therapy with 29 having negative circumferential margins.

In the re-audit, the CRM+ve rate was only 3% (1 out of 37) for all surgery alone patients (i.e. including the three patients not discussed). There was also a 3% CRM+ve rate (2 out of 64) in all resected patients discussed at MDT including those resected following preoperative neoadjuvant therapy. If the three patients with irresectable disease are included together with the patients who died during preoperative neoadjuvant therapy, the cumulative CRM+ve rate is 7% (5 out of 71) in discussed patients.

The CRM+ve rate for all re-audit patients who underwent MDT discussion following an MRI (7%) is comparable to the original audit of 8% (16 out of 197) and significantly lower than national figures of <20% (2004) (*P*<0.001). Overall effective CRM+ve rates for all potentially curable patients – including those patients with unresectable disease after preoperative neoadjuvant therapy and those not discussed at MDT – was reduced from 12.5% (32 out of 259) in the original audit to 7% (5 out of 71) in the re-audit.

## DISCUSSION

The success of rectal cancer treatment has traditionally been measured in terms of local recurrence rates and predicted survival curves. However, definitions of local recurrence vary between series (Anwar *et al*, 2001) and the recent demonstration of the role of preoperative therapy has been directed at improving these outcomes. The introduction of TME has reduced local recurrence rates and these results have now been replicated (MacFarlane *et al*, 1993; Havenga *et al*, 1999; Cecil *et al*, 2004).

The debate over preoperative *vs* postoperative radiotherapy has been resolved (Sauer *et al*, 2004) and neoadjuvant therapy with either chemoradiation or long course chemotherapy has emerged as an effective treatment in the downstaging/downsizing of locally advanced tumours (Chau *et al*, 2003; Rodel *et al*, 2003). This has resulted in a greater proportion of patients being considered for curative resection (Janjan *et al*, 1999; Chau *et al*, 2003). In our series, 60 out of 259 potentially curative cases (23%) would have traditionally been deemed inoperable based upon local invasion and tumour fixity, but 45 (75%) of these underwent a CRM−ve resection following preoperative neoadjuvant therapy. Although these results are encouraging, long-term follow-up of these patients is required to determine the success of the strategy in maintaining local and systemic control. Some evidence addressing the issue of long-term follow-up is already available from a multicentre retrospective study of 541 patients with locally advanced rectal cancer undergoing preoperative CRT than surgical resection, with 41% of CRM+ve patients (48 out of 115) developing local recurrence as compared to only 7% (31 out of 426) CRM−ve patients after 17–37 months (Sebag-Montefiore *et al*, 2005).

From review of the notes of patients in the original audit without MDT discussion of MRI, it appears there was a combination of factors accounting for their suboptimal management. Twenty of these cases (32%) underwent an MRI, which was not reviewed preoperatively at an MDT – subsequent review of these images demonstrates that poor prognostic features could be identified in 10 cases (50%). Review of the original reports indicates understaging in some cases and could reflect the learning curve of MRI reporting for rectal cancer in less experienced radiologists. This highlights the need for MDT discussion and review of the films to aid identification of diagnostically challenging cases. In other cases, the surgeon deemed the tumour resectable on clinical examination but was concerned about impending obstruction and therefore, failed to wait for the MRI report before proceeding with surgery.

In the original audit, only 13% of patients were deemed palliative as compared to 24% on re-audit. The identification of a higher proportion of palliative patients in the re-audit may reflect the introduction of the directive that all patients should be discussed at MDT with more thorough staging and assessment of irresectable metastatic disease and medical infirmity.

Circumferential resection margin rates are becoming established as a short-term outcome measure of local recurrence, distant metastases and poor survival (Adam *et al*, 1994; Hall *et al*, 1998; Birbeck *et al*, 2002; Wibe *et al*, 2002). Routine histopathological assessment of the CRM gives objective evidence of the effectiveness of treatment of rectal cancer (Birbeck *et al*, 2002). National randomised controlled therapeutic trials are using the CRM rate as a short-term outcome measure (Marijnen *et al*, 2003). In the literature, resections can be classified as ‘curative’ or ‘palliative’ without any obvious preoperative staging criteria or any standardisation of the criteria. Circumferential resection margin positive rates of 25% have been reported in series of selected ‘curative’ resections (Adam *et al*, 1994). With the development of MRI, it is now possible to accurately predict tumour stage and other prognostic features such as nodal disease, depth of extramural spread and the presence of vascular invasion (Brown *et al*, 1999, 2003; Botterill *et al*, 2001). Therefore, curative intent can be determined preoperatively.

A reduction in CRM positivity should be the goal of colorectal MDTs. Our CRM+ve/irresectable rate of 8% (95% CI=3.9–11.4%) for this unselected and consecutive series of patients having MDT discussion of MRI is significantly better than the accepted national standards of <20% (2004). The guidelines specify CRM+ve rates of <20% in potentially curative cases but excluding patients undergoing LRT (2004). This rate was comparable (7.4%) in the re-audit. However, the overall CRM+ve rate for all potentially curative patients including those without MDT discussion of MRI in the original audit was 12.5%. This was reduced to 7% in the re-audit after mandating preoperative MRI-based MDT discussion of all rectal cancer patients. Our MDT network strategy is based on identifying those patients curable by surgery alone and intensifying treatment in those patients at risk of local and systemic failure. Our results demonstrate the effectiveness of an MRI-based MDT discussion in implementing preoperative treatment strategies. Discussion and demonstration of the MRI findings in the presence of all the MDT members appears more useful than issuing a standard report in which subtleties may be missed.

In conclusion, it appears that the CRM+ve rate is reducible, but only in the presence of robust MRI staging, preoperative MDT discussion of all the staging investigations, optimal surgery, the availability of effective preoperative therapies and standardised histopathology reporting with comprehensive data collection.

## Figures and Tables

**Table 1:** Locally agreed treatment policy for rectal cancer within the MDT

**Treatment group**	**MRI features**	**Treatment strategy**
1	T1–T2/T3 <5 mm, N0/N1, predicted CRM−ve	Surgery alone (TME)
2	T3≥5 mm/T4, N2, predicted CRM−ve	Preoperative chemoradiotherapy^a^
3	Predicted CRM+ve^b^	Preoperative chemoradiotherapy^a^

Our local treatment stratification was based on evidence of 5-year survival rates of 85% for rectal cancer patients with T3 tumours <5 mm depth of extramural spread *vs* 54% survival for cases with T3 ⩾5 mm spread (Merkel *et al*, 2001).

MDT=multidisciplinary team; MRI=magnetic resonance imaging; TME=total mesorectal excision.

a Patients with contraindications to the use of systemic chemotherapy were offered LRT as a preoperative treatment.

b Defined as tumour within 1 mm of the mesorectal fascia or >T2 tumour arising from below the level of the origin of the levator muscles (Figure 1).

**Table 2:** Comparison of MRI predicted stage and final histological stage in discussed patients

**MRI staging of patients with MDT discussion of MRI**	
T1–T2, T3 <5 mm, N0-1, CRM−ve	116 (59%)
T3 ⩾5 mm, T4, N2, CRM−ve	21 (11%)
CRM+ve	60 (30%)
Total	197
	

**Final histological staging in resected patients with MDT discussion of MRI**	
T1–2, T3<5 mm, N0-1, CRM−ve	152 (77%)
T3⩾5 mm, T4, N2, CRM−ve	26 (13%)
CRM+ve	4 (2%)
Unresected patients	15 (8%)
Total	197

MDT=multidisciplinary team; MRI=magnetic resonance imaging.

**Table 3:** Histopathological staging of patients who did not undergo MDT discussion of MRI

**Histopathology of patients undergoing surgery alone without MDT discussion of MRI**	
T1–T2, T3 <5 mm, N0-1, CRM−ve	30 (48%)
T3⩾5 mm, T4, N2, CRM−ve	16 (26%)
CRM+ve	16 (26%)
Total	62

MDT=multidisciplinary team; MRI=magnetic resonance imaging.

## Appendix A

### MEMBERS OF RMH COLORECTAL MULTIDISCIPLINARY TEAM

*Mayday University Hospital*: Mr I Swift, Mr AM Abulafi, Ms S Burton, Mr MLC Stellakis, Dr Blake, Dr N Bees, Dr A Arnaout, Mrs A Stewart.

*Epsom & St Heliers NHS Trust*: Mr MA Raja, Mr P Toomey, Mr N Bett, Mr S Farhat, Mr IR Daniels, Dr LN Temple, Dr CD George, Mrs S Woodward.

*Royal Marsden Hospital*: Professor D Cunningham, Dr D Tait, Dr G Brown, Dr A Wotherspoon, Dr G Chong, Dr AR Norman
